# Supplementary material for: AffyMAPSDetector: a software tool to characterize Affymetrix GeneChip™ expression arrays with respect to SNPs
Source: BMC Bioinformatics. 2007 Jul 30;8:276. doi: 10.1186/1471-2105-8-276 (PMC1959249; doi:10.1186/1471-2105-8-276)
Supplement: Additional file 13 — AffyMAPSDetector v1 source code. AffyMAPSDetector source code. [file 1471-2105-8-276-S13.zip › AffyMAPSDetector_v1_src/doc/constant-values.html]

Constant Field Values


|  |  |  |  |
| --- | --- | --- | --- |
| **Overview** \| Package \| Class \| Use \| **Tree** \| **Deprecated** \| **Index** \| **Help** | | |  |
| PREV   NEXT | **FRAMES**    **NO FRAMES**     **All Classes** |  |  |


---


# Constant Field Values


---

**Contents**

- affychip.snpdetector.\*- jwf.\*

**affychip.snpdetector.\***

|  |  |  |
| --- | --- | --- |
| **affychip.snpdetector.Constant** | | |
| `public static final java.lang.String` | `ADD_TO_FILTERED_ROWS` | `"Add back to filtered probes"` |
| `public static final java.lang.String` | `ADD_TO_REMOVED_ROWS` | `"Add to removed probes"` |
| `public static final java.lang.String` | `GENE_ANNOT` | `"Gene Description"` |
| `public static final java.lang.String` | `GENE_ANNOT_FIELD_INDEX` | `"geneAnnotField"` |
| `public static final java.lang.String` | `GENE_IDENTIFIER_FIELD_INDEX` | `"gifIndex"` |
| `public static final java.lang.String` | `GENE_INFO_FILE` | `"geneInfoFile"` |
| `public static final java.lang.String` | `GENE_INFO_FILE_SEPARATOR` | `"gifSeparator"` |
| `public static final java.lang.String` | `GENE_INFO_HEADERS` | `"geneInfoHeaders"` |
| `public static final java.lang.String` | `GENE_INFO_PROBESET_ID_FIELD_INDEX` | `"gipsifIndex"` |
| `public static final java.lang.String` | `HAS_SNPS_FILENAME` | `"hasSnpsFilename"` |
| `public static final java.lang.String` | `HEAT_MAP` | `"Heat Map"` |
| `public static final java.lang.String` | `LOCUS_LINK_FIELD_INDEX` | `"locusLinkFieldIndex"` |
| `public static final java.lang.String` | `MM_POS_FIELD_INDEX` | `"mmPosFieldIndex"` |
| `public static final java.lang.String` | `NO_LOCUS_LINK_FILENAME` | `"noLocusLinkFilename"` |
| `public static final java.lang.String` | `NO_SNPS_FILENAME` | `"noSnpsFilename"` |
| `public static final double` | `ONE_MEG` | `1000000.0d` |
| `public static final java.lang.String` | `ORGANISM` | `"organism"` |
| `public static final java.lang.String` | `PM_MM_RATIO_DATA` | `"PM/MM Ratio Data"` |
| `public static final java.lang.String` | `PROBE_INFO_FILE` | `"probeInfoFile"` |
| `public static final java.lang.String` | `PROBE_INFO_FILE_SEPARATOR` | `"pifSeparator"` |
| `public static final java.lang.String` | `PROBE_INFO_HEADERS` | `"probeInfoHeaders"` |
| `public static final java.lang.String` | `PROBE_INFO_PROBESET_ID_FIELD_INDEX` | `"pipsifIndex"` |
| `public static final java.lang.String` | `PROBE_SEQ_FIELD_INDEX` | `"probeSeqFieldIndex"` |
| `public static final java.lang.String` | `VIEW_FILTERED_PROBES` | `"Filtered Probes"` |
| `public static final java.lang.String` | `VIEW_REMOVED_RPOBES` | `"Removed Probes"` |
| `public static final java.lang.String` | `WEB_GENE_INFO_FILENAME` | `"webGeneInfoFilename"` |
| `public static final java.lang.String` | `WEB_SNP_INFO_FILEMANE` | `"webSnpInfoFilename"` |
| `public static final java.lang.String` | `X_POS_FIELD_INDEX` | `"xPosFieldIndex"` |
| `public static final java.lang.String` | `Y_POS_FIELD_INDEX` | `"yPosFieldIndex"` |

|  |  |  |
| --- | --- | --- |
| **affychip.snpdetector.model.SnpDetector** | | |
| `public static final java.lang.String` | `findLocusLinkForAccessionNo` | `"http://www.ncbi.nih.gov/entrez/query.fcgi?db=gene&term="` |
| `public static final java.lang.String` | `ncbiGeneAccessionNoSearchUrl` | `"http://www.ncbi.nlm.nih.gov/entrez/query.fcgi?cmd=Search&db=Nucleotide&term="` |
| `public static final java.lang.String` | `ncbiNucleotideDbUrl` | `"http://www.ncbi.nlm.nih.gov/entrez/batchseq.cgi?txt=on&db=nucleotide&view=fasta&SendTo=t&list_uids="` |
| `public static final java.lang.String` | `ncbiRefSnpClusterUrl` | `"http://www.ncbi.nlm.nih.gov/SNP/snp_ref.cgi?rs="` |
| `public static final java.lang.String` | `ncbiSnpDbUrl` | `"http://www.ncbi.nlm.nih.gov/SNP/snp_ref.cgi?type=GenBank&geneId="` |

|  |  |  |
| --- | --- | --- |
| **affychip.snpdetector.util.PmMmHeatMapViewer** | | |
| `public static final java.lang.String` | `E` | `"Expr:\t"` |
| `public static final java.lang.String` | `G` | `"Gene:\t"` |
| `public static final java.lang.String` | `S` | `"Sample:\t"` |

**jwf.\***

|  |  |  |
| --- | --- | --- |
| **jwf.Wizard** | | |
| `public static final java.lang.String` | `BACK_I18N` | `"BACK_I18N"` |
| `public static final java.lang.String` | `CANCEL_I18N` | `"CANCEL_I18N"` |
| `public static final java.lang.String` | `FINISH_I18N` | `"FINISH_I18N"` |
| `public static final java.lang.String` | `HELP_I18N` | `"HELP_I18N"` |
| `public static final java.lang.String` | `NEXT_I18N` | `"NEXT_I18N"` |

|  |  |  |
| --- | --- | --- |
| **jwf.WizardUtils** | | |
| `public static final int` | `MAX_LABEL_WIDTH` | `40` |

---


|  |  |  |  |
| --- | --- | --- | --- |
| **Overview** \| Package \| Class \| Use \| **Tree** \| **Deprecated** \| **Index** \| **Help** | | |  |
| PREV   NEXT | **FRAMES**    **NO FRAMES**     **All Classes** |  |  |


---
